# The Effects of Applied Behavior Analysis on Verbal Behavior With Autistic Individuals Using the Verbal Behavior Milestones Assessment and Placement Program (VBMAPP) and the Assessment of Basic Language and Learning Skills (ABLLS)

**DOI:** 10.7759/cureus.57041

**Published:** 2024-03-27

**Authors:** Tami Peterson, Jessica Dodson, Tiffany Hosey, Robert Sherwin, Frederick Strale,

**Affiliations:** 1 Hyperbaric Oxygen Therapy, The Oxford Center, Brighton, USA; 2 Applied Behavior Analysis, The Oxford Center, Brighton, USA; 3 Hyperbaric Oxygen Therapy, Wayne State University School of Medicine, Detroit, USA; 4 Biostatistics, The Oxford Center, Brighton, USA

**Keywords:** vbmapp, skinner, ablls, autism and verbal behavior, patient-centered outcomes research, verbal behavior milestones, applied behavior analysis, autism spectrum disorder (asd)

## Abstract

Introduction

Applied behavior analysis (ABA) is a fundamental practice-based intervention for treating autism spectrum disorder (ASD). Few studies have directly measured and evaluated the effects of ABA on verbal behaviors, mainly using the Verbal Behavior Milestones Assessment and Placement Program (VBMAPP) and the Assessment of Basic Language and Learning Skills (ABLLS) as outcome measures. This study aims to fill this gap by examining the relationship between ABA interventions and the enhancement of verbal skills, as measured by the VBMAPP and the ABLLS, in a convenience sample of individuals with ASD.

Materials and methods

At The Oxford Centers (TOCs) in Brighton and Troy, Michigan, USA, 33 individuals with autism received treatment from January 2018 to July 2021, spanning 43 months. A pretest-posttest design was employed to retrospectively examine any impacts between ABA interventions and alterations in verbal scores among individuals with ASD. Depending on developmental age, all subjects underwent two verbal assessments with a six-month interval in-between. Twelve children were administered the VBMAPP, while 21 were given the ABLLS.

Results

Paired t-tests for pretest and posttest VBMAPP subscales resulted in statistically significant effects (p<0.05) for (VBMAPP - Mand), (VBMAPP - Tact), (VBMAPP - Listener Responding), (VBMAPP - Visual Perceptual Skills and Matching-to-Sample), (VBMAPP -Independent Play), (VBMAPP - Social Play), (VBMAPP - Motor Imitation), (VBMAPP - Spontaneous Vocalization), (VBMAPP - Intraverbal), (VBMAPP - Group Behavior), and (VBMAPP - Linguistic Structure). As measured by Cohen's d, effect sizes were moderate to mostly high (-0.623 to -1.688). There were non-significant results (p>0.05) for (VBMAPP - Listener Responding by Feature, Function, and Class) and (VBMAPP - Echoic).

Paired t-tests for pretest and posttest ABLLS subscales resulted in statistically significant effects (p<.05) for all ABLLS scales: (ABLLS - Receptive Language), (ABLLS - Requests), (ABLLS - Labeling), (ABLLS - Intraverbals), (ABLLS - Spontaneous Vocalizations), (ABLLS - Syntax Grammar), (ABLLS - Social Interactions), and (ABLLS - Generalized Responding). As measured by Cohen's d, effect sizes were moderate to mostly high (-0.656 to -1.372).

Conclusions

The administration of ABA treatments had a noteworthy influence, with statistically significant impacts on improving verbal behaviors on 11 of the 13 VBMAPP scales and all of the ABLLS scales. As measured by Cohen's d, effect sizes were moderate to high for both scales. These findings underscore the importance and effectiveness of ABA interventions in enhancing verbal skills in children with ASD. However, it's crucial to note that further confirmatory studies are required to verify the reliability of these original findings, emphasizing the ongoing need for research in this field.

## Introduction

The verbal operant is considered a suitable unit of analysis with implications for various components of an applied behavior analysis (ABA) intervention program aimed at treating individuals with autism spectrum disorder (ASD). Many individuals with autism may face challenges or be incapable of communicating using speech or language skills, and some may have limited speaking abilities, leading to difficulties in expressing themselves and understanding others [1,2].

Specific individuals with ASD may possess advanced vocabulary skills and engage in detailed discussions on various subjects. Still, they may find it challenging to use verbal behavior in social situations, such as starting conversations, greeting others, and making requests [2]. They may also struggle with non-verbal cues like facial expressions, eye contact, and body language. Some individuals with autism may echo others' words (a phenomenon known as echolalia) immediately or later. Common literacy impairments in these individuals include reading, writing, spelling, and comprehension, which can impact their academic performance, access to information, and writing skills [3].

Individuals with ASD may also exhibit verbal deficits linked to sensory issues. They might be overly sensitive to sounds and loud noises, which could lead them to avoid speech or make them unaware of their voice's volume [3]. There are numerous behavioral interventions for ASD, including behavioral, developmental, educational, social-relational, pharmacological, psychological, and complementary or alternative interventions. Regrettably, despite early diagnosis and intensive therapy, individuals with ASD continue to face significant challenges in social and verbal interaction, academics, and overall life experience [4]. Jayakumar et al. [5] underscored the importance of early, intensive ABA treatment for individuals with autism, noting a positive trend in all levels of verbal behavior as measured by the Verbal Behavior Milestones Assessment and Placement Program (VBMAPP) over time, despite diverse initial presentations [1,2,4,5].

Sundberg and Partington [6] highlighted Skinner’s [7] principles of verbal behavior in their approach to teaching language to individuals with autism. Language is understood through verbal behavior concepts, and these verbal behaviors are systematically taught to children. The process starts with mand training to enhance an individual's frequency and variety of mands.

Early education of individuals with autism prioritizes mand training because it focuses on enhancing spontaneous and functional communication. Mand training exposes the individual with autism to a rich environment filled with preferred stimuli and offers frequent reinforcement for communicative behavior. Mands (requests) are shaped, building on behaviors already in the individual's repertoire. Prompting and modeling can enhance the frequency and complexity of mands. Progress is tracked by recording the type and frequency of mands within specific time intervals. Once a solid range of regularly used mands is established, instruction can shift focus to other language functions such as tacting [6].

Natural Environment Training (NET) pertains to the structure and context of teaching verbal behavior. NET, as described by Sundberg and Partington [6], is based on the Natural Language Paradigm (NLP). In NET and NLP, the individual selects the stimulus items, which are frequently varied and functionally relevant to the interaction. This allows teaching exchanges within naturally occurring interactions, leveraging an individual's interest and momentary motivation. Research has shown that autistic individuals can acquire language and communication skills using NET and NLP and that using a natural environment may decrease disruptive behavior [6-9,10].

Mand training and NET are sometimes contrasted with discrete trial instruction (DTI), typically seen as instructor-directed, more repetitive, and involving fewer natural contingencies and reinforcements. However, DTI can be incorporated within more natural environments and teaching interactions. The structured sequence of discriminative stimuli (SD), response, and consequence can be readily embedded within patient-directed activities, including varied materials and relevant, functional outcomes [6-9].

Harris and Delmolino [10] emphasized the need to subject these methods to long-term outcome studies that have documented the benefits of discrete trial teaching in its more traditional context [10,11]. Peterson et al. [1,12-14] outlined outcomes and provided further evidence for the ongoing impact of ABA using discrete trial training (DTT) and mass trials within a naturalistic environment with individuals with autism during various snapshot periods. Statistically significant mean differences in target behaviors were determined across two-week intervals. Studies are scarce in the literature measuring and assessing the direct impacts of ABA on verbal behaviors, especially using the VBMAPP [1,2] and the Assessment of Basic Language and Learning Skills (ABLLS) [1,14] as outcome instruments. 

Objectives 

The main goal and outcome of interest of this study are to assess any impact between the mixed model ABA interventions, namely, DTI, mass trials, and NET, and the enhancement of verbal skills [1], as gauged by the VBMAPP [2] and the ABLLS [15], before and after the intervention, in a convenience sample of individuals with ASD. It is hypothesized that the combination of DTT, mass trials, and naturalistic environment training will significantly improve autistic individuals' verbal behaviors as measured by VBMAPP and ABLLS.

## Materials and methods

Study setting and participants 

Between January 2018 and July 2021 (43 months), 33 individuals with autism were attended to, provided treatment, and evaluated at The Oxford Centers (TOCs) located in Brighton and Troy, Michigan, USA. TOCs are outpatient clinics offering various clinical services for multiple conditions, including ASD [1].

Data collection

Data for the study were retrospectively gathered from electronic medical records by skilled research assistants, focusing on a cohort of individuals with autism who underwent ABA treatment. Three distinct "non-author" board-certified behavior analysts (BCBAs) administered and collected the original pretest and posttest data for VBMAPP (n=12) and ABLLS (n=21). The generation and reporting of the manuscript adhered to a hybrid of the Consolidated Standards of Reporting Trials (CONSORT) and Strengthening the Reporting of Observational Studies in Epidemiology (STROBE) guidelines. Records of individual cohorts aged two to 17 years diagnosed with ASD were examined for inclusion in the study. Matched pairs (pretest-posttest) were formed, ensuring that the children's cohorts were identical in every aspect except for the ABA intervention. The individual cohorts acted as their own controls in the pretest-posttest, thereby minimizing potential bias from extraneous variables [1,12-14]. 

ABA intervention

All participants underwent ABA treatment, receiving at least 25 hours of ABA therapy per week. ABA is a one-on-one therapeutic approach that enhances the skills of individuals with ASD, enabling them to thrive in their homes, schools, and communities through natural environment teaching and discrete trial teaching. All individuals with autism were attended to and treated with a mixed methods approach to ABA, utilizing DTT, mass trials, and naturalistic environment training treatment modalities. Before training, each child cohort was provided with a treatment plan crafted by one of eight BCBAs tailored to the individual's needs and objectives. Each individual was assigned to one of 83 behavioral technicians, and a team of three to five behavioral technicians administered the ABA treatments. Suitable materials were chosen and arranged in rooms where individual DTT and mass trials occurred or in a naturalistic setting where the participant engaged with others and encountered functional and meaningful real-world situations. Each behavioral technician was assigned to a different participant daily, delivering, on average, four to seven hours of treatment per day, amounting to at least 25 hours a week [1,12-14].

Outcome measures 

A BCBA conducted the initial (pretest) assessment based on the individual's developmental age using either the VBMAPP or ABLLS. Initial goals were established and subsequently reassessed after a period of observation. The control for potential confounding variables was achieved through matched pairs (pretest-posttest), with each cohort acting as its own control [1,12-14].

*VBMAPP* *and VBMAPP Scales*

The BCBA evaluated individual cohorts (n=12) at the pretest and posttest stages. Each subject was tested on various verbal milestone domains of the VBMAPP, including Mand, Tact, Listener Responding, Visual Perceptual Skills and Matching-to-Sample, Independent Play, Social Play, Motor Imitation, Echoic, Spontaneous Vocalization, Listener Responding by Feature, Function, and Class, Intraverbals, Group Behavior, and Linguistic Structure. These skills are crucial for the development of language and social skills. Each subject was observed, prompted, and assigned a pretest and posttest rating on a five-point Likert scale grid based on response behaviors recorded by the BCBA. A higher score on the milestone subscale indicates better progress for the individual cohort [2]. Cronbach's alpha on VBMAPP for this sample was r=0.972 [16].

Mand: It involves the speaker asking for what they want. For example, a teacher might ask a student, "What do you want?" and the student responds with "juice." In this scenario, the student has effectively requested a drink of juice. Developing manding skills is essential because it empowers the children to advocate for their wants and needs [3].

Tact: It refers to naming something. In the context of the VBMAPP, tacts involve labeling or identifying objects, actions, or events in the environment. For instance, an individual points to a car and says, "car." An individual sees a flower and verbally identifies it as a "flower." Developing tacts is crucial because they enable individuals to express their understanding of the world around them. It's like saying what they see, hear, or touch [3].

Listener Responding (LR): It assesses an individual's ability to respond to verbal stimuli from others. Essentially, it evaluates how well someone comprehends and reacts to spoken language. Listening is a critical skill for effective communication and social interactions. Examples are when a teacher says, "Point to the red ball," and the student correctly points to the red ball; that's the listener responding. Following instructions like "Give me the blue crayon" or "Show me the picture of a cat" also fall under this category [3].

Visual Perceptual Skills and Matching-to-Sample (VPMTS): It assesses an individual's ability to perform visual discrimination tasks. It involves matching non-identical items, sorting by size, associating items, categorizing objects, completing patterns, and following sequential order. Visual perceptual skills are fundamental for understanding and interacting with the environment. Matching-to-sample tasks help individuals recognize similarities and differences between objects or pictures [3].

Independent Play: It is an individual's ability to engage in play activities without direct interaction or guidance from others. It involves playing alone, exploring toys, and entertaining oneself. The importance is autonomy. Independent play fosters an individual's independence and self-sufficiency. It allows them to explore their environment and develop creativity and social skills. While independent, individuals learn to manage their emotions, solve problems, and entertain themselves. These skills contribute to their overall social development [3].

Social Play: It assesses an individual's ability to play and interact with others. It focuses on social behaviors, cooperative play, and communication during shared activities. Social play is essential for developing social skills, understanding social cues, and building relationships. It involves turn-taking, joint attention, sharing, and understanding social rules [3].

Motor Imitation: It refers to an individual's ability to imitate physical movements demonstrated by others. It involves copying actions, gestures, or motor patterns observed in the environment. Motor imitation is crucial for learning and social interaction. It allows individuals to learn by following and replicating movements made by peers, caregivers, or teachers [3].

Echoic: It assesses an individual's ability to repeat or echo auditory stimuli. It involves imitating spoken words or phrases after hearing them from someone else. Echoic behavior is a crucial building block for language development. It allows individuals to learn and practice verbal skills by imitating sounds and words [3].

Spontaneous Vocalization: It refers to an individual's ability to produce verbal sounds or words without direct prompting or imitation. It involves spontaneously using language to express thoughts, feelings, or needs. Spontaneous vocalization is a critical milestone for language development. It allows individuals to communicate independently and share their experiences with others [3].

Listener Responding by Feature, Function, and Class (LRFFC): It is an advanced type of listener-responding behavior that focuses on identifying objects based on specific characteristics. It involves recognizing objects by their associated features, functions, or categories (classes). When students are asked to "Touch something you eat" (function), they might touch food items. LRFFC helps children develop a deeper understanding of objects in their environment. It teaches them adjectives and verbs related to different objects [3].

Intraverbals: These refer to verbal behaviors where an individual responds to verbal stimuli from others without direct physical cues. Unlike echoing or repeating, intraverbals involve generating novel responses based on context. Examples are completing sentences (e.g., "Twinkle, twinkle, little ____") and answering questions (e.g., "What's your favorite color?") [3].

Group Behavior: It assesses an individual's ability to engage in appropriate behaviors within a group or classroom setting. These behaviors include following group instructions, participating in group activities, and demonstrating social skills. Group behavior skills are essential for successful inclusion in educational and social environments. They allow individuals to interact effectively with peers, teachers, and classmates [3].

Linguistic Structure: It assesses an individual's language complexity and grammatical skills. It focuses on various linguistic elements, including sentence structure, verb tenses, pronouns, and syntax. Examples are an individual saying, "I want juice" (using subject-verb-object structure), which demonstrates the linguistic structure and correctly using pronouns (e.g., "he," "she," "they") in sentences [3].

ABLLS and ABLLS Scales

Individual cohorts (n=21) were evaluated on a grid by a BCBA at the pretest and posttest stages. Each cohort was tested on various behavioral milestone subscales, including Receptive Language, Requests, Labeling, Intraverbals, Spontaneous Vocalizations, Syntax Grammar, Social Interaction, and Generalized Responding [1,14]. Cronbach's alpha on ABLLS for this sample was r=0.851 [1,16].

Receptive Language: It refers to an individual's ability to understand and process spoken or written language. It involves comprehending words, sentences, and instructions. Teachers and therapists use strategies such as modeling and clearly presenting language examples, prompting, providing cues to guide correct responses, and reinforcement, rewarding accurate receptive language skills [15].

Requests: These assess an individual's ability to make requests or ask for desired items or actions. It involves using language to express needs, wants, and preferences. Teachers and therapists use strategies such as modeling, demonstrating how to make requests, prompting, providing cues or assistance to encourage accurate requests and reinforcement, and rewarding successful requests [15]. 

Labeling: It assesses an individual's ability to name or label objects, actions, and concepts. It involves using expressive language to identify and describe items in the environment. Teachers and therapists use strategies such as modeling, clearly naming objects and providing examples, expanding responses, encouraging more detailed descriptions and reinforcement, and rewarding accurate labeling [15].

Intraverbals: These refer to verbal behaviors where an individual responds to verbal stimuli from others without direct physical cues. Unlike echoing or repeating, intraverbals involve generating novel responses based on context. Examples include completing sentences (e.g., "Twinkle, twinkle, little ____"), answering questions (e.g., "What's your favorite color?"), and engaging in conversations (e.g., discussing a topic). Teachers and therapists use various strategies to teach intraverbals: prompting, providing cues or prompts to evoke appropriate responses, expanding responses, encouraging longer and more detailed answers, and reinforcement, rewarding accurate intraverbal reactions [15].

Spontaneous Vocalizations: These refer to an individual's ability to produce verbal sounds or words without direct prompting or imitation. It involves spontaneously using language to express thoughts, feelings, or needs. Teachers and therapists use strategies such as creating opportunities, namely, providing situations, where the individual can express themselves verbally, expanding utterances, encouraging longer and more descriptive vocalizations, and reinforcement, rewarding spontaneous vocalizations [15].

Syntax Grammar: It assesses an individual's ability to use correct sentence structure and grammar. It focuses on understanding and producing grammatically accurate sentences. Teachers and therapists use strategies such as modeling correct sentences, namely, providing examples of proper sentence structure, expanding responses, encouraging longer and more grammatically complete answers, and reinforcement, rewarding accurate syntax usage [15].

Social Interactions: These assess an individual's ability to engage in socially appropriate behaviors and interactions. It focuses on skills related to communication, joint attention, turn-taking, and understanding social cues. Teachers and therapists use strategies such as modeling, demonstrating appropriate social behaviors and social stories, creating narratives to teach social rules and expectations, and reinforcement, namely, rewarding positive social interactions [15].

Generalized Responding: It refers to an individual's ability to demonstrate a skill or behavior across different settings, people, and materials. It involves applying learned skills in various contexts beyond the initial teaching environment. The importance of generalized responding ensures that skills are not limited to specific situations. It promotes flexibility and adaptability in using acquired knowledge [15].

Power analysis

A retrospective power analysis was performed using G*Power 3.1 [1,17]. The analysis suggested that a total of 23 participants would be necessary to show a high group effect size (0.80) for the difference between two dependent means (matched pairs) with a nominal alpha (α)=0.05 using a two-tailed paired t-test, achieving a power of 0.956. Given these parameters from the power analysis, the current study with n=33 participants is likely to meet an acceptable sample size criterion [1].

Data analyses 

All descriptive and inferential data analyses were conducted using IBM SPSS Statistics for Windows, V. 29.0 (released in 2022 by IBM Corp., Armonk, NY, USA) [18]. Summaries of demographics and baseline characteristics were provided for all subjects. Summary statistics were generated for all continuous variables, such as the number of subjects, mean and standard deviation, and median and range. These variables included age, months from the baseline (pretest) verbal test to the post-baseline verbal test, age VBMAPP, and age ABLLS [1]. For all categorical variables, such as race/ethnicity and autism severity level, the number and percentage of subjects within each category were presented. The change from baseline in the VBMAPP and ABLLS scores was summarized. The nominal alpha was set at 0.05, two-tailed. Statistical significance was declared for p<0.05. All statistical results were reported in text and table formats [1].

Interobserver reliability

VBMAPP

For the VBMAPP, a two-way random effects model was computed where people's effects and measures effects were kept random. We used the intraclass correlation coefficient (ICC) two-way random effects model (2), which is used when multiple measurements are made from each averaged rater. The ICC (2) value was 0.719 (95% CI: 0.438-0.901), indicating a good agreement between the raters. This value was more significant than the average Pearson r=0.193, suggesting that the ICC (2) was more sensitive to the variability among raters and measurements. Cronbach's alpha for the pretest-posttest differences was r=0.752, indicating good internal consistency and reliability.

ABLLS

For the ABLLS, a two-way random effects model was computed where people's effects and measures effects were also kept random. We used the ICC two-way random effects model (2), which is used when multiple measurements are made from each averaged rater. The ICC (2) value was 0.786 (95% CI: 0.607-0.900), indicating a good agreement between the raters. This value was more significant than the average Pearson r=0.404, suggesting that the ICC (2) was more sensitive to the variability among raters and measurements. Cronbach's alpha for the pretest-posttest differences was r=0.851, indicating good internal consistency reliability.

Independent ethics committee

Consent was obtained or waived by all participants in this study. This research study retrospectively used data collected from chart reviews for clinical purposes. The study was submitted to the WIRB-Copernicus Group (WCG®IRB) for review and was granted an exemption (#1-1703366-1). The authors declare that this research investigation involves minimal risk and complies with the Belmont Report Regulations 45 CFR 46 2018 Requirements (2018 Common Rule) Section 46 Subpart A Basic HHS Policy for Protection of Human Research Subjects, 46.104 Exempt Research Paragraph d (1), (2), and (2) ii, and 46.117 Documentation of Informed Consent Paragraph c (1) (ii). This study also conformed to the 1964 Declaration of Helsinki guidelines.

## Results

Descriptive statistics

Demographics

For the sample of 33 individuals with autism, the age had a mean of 6.55 and a standard deviation of 3.58, the median was 5, the minimum was 2, and the maximum was 16. For the sample of 12 individuals with autism who were measured with VBMAPP, the age had a mean of 4.08 and a standard deviation of 1.08, the median was 5, the minimum was 2, and the maximum was 16. For the sample of 21 individuals with autism who were measured with ABLLS, the age had a mean of 7.96 and a standard deviation of 3.76, the median was 7, the minimum was 4, and the maximum was 16. There were 27 males (81.8%) and six females (18.2%). There were 23 Caucasians (69.7%), zero Asians (0.0%), three Hispanics (9.1%), two Middle Eastern (6.1%), zero African Americans (0.0%), one Native American (3.0%), two Middle Easterners (6.1%), and three unspecified (9.1%). Nineteen (57.6%) individuals had an autism severity level of 3, 11 (33.3%) had an autism severity level of 2, and three (9.1%) had an autism severity level of 1. The period from the baseline verbal pretest to the post-baseline verbal test had a mean of 5.8 months and a standard deviation of 0.73. The median duration was six months, with a minimum of four months and a maximum of eight months.

Inferential statistics

General paired t-test results for both VBMAPP and ABLLS pretest and posttest were as follows: Pair 1 (VBMAPP - Mand): t(11)=-5.745, p<0.001, d=-1.658; Pair 2 (VBMAPP - Tact): t(11)=-3.843, p=0.003, d=-1.109; Pair 3 (VBMAPP - Listener Responding): t(11)=-4.416, p-0.001, d=-1.275; Pair 4 (VBMAPP - Visual Perceptual Skills and Matching-to-Sample): t(11)=-4.363, p=0.001, d=-1.259; Pair 5 (VBMAPP - Independent Play): t(11)=-3.370, p<0.006, d=-0.973; Pair 6 (VBMAPP - Social Play): t(11)=-4.733, p<0.001, d=-1.366; Pair 7 (VBMAPP - Motor Imitation): t(11)=-4.661, p<0.001, d=-1.346; Pair 8 (VBMAPP - Echoic): t(11)=-2.159, p<0.054, d=-0.623; Pair 9 (VBMAPP - Spontaneous Vocalization): t(11)=-3.137, p<0.009, d=-0.906; Pair 10 (VBMAPP Listener Responding by Feature, Function, and Class): t(11)=-1.688, p=0.119, d=-0.487; Pair 11 (VBMAPP - Intraverbal): t(11)=-3.251, p<0.008, d=-0.938; Pair 12 (VBMAPP - Group Behavior): t(11)=-2.960, p<0.013, d=-0.855; Pair 13 (VBMAPP - Linguistic Structure): t(11)=-2.385, p=0.036, d=-0.689; Pair 14 (ABLLS Receptive Language): t(20)=-6.285, p<0.001, d=-1.372; Pair 15 (ABLLS - Requests): t(20)=-5.359, p<0.001, d=-1.169; Pair 16 (ABLLS - Labeling): t(20)=-5.411, p<0.001, d=-1.181; Pair 17 (ABLLS - Intraverbals): t(20)=-4.244, p<0.001, d=-0.926; Pair 18 (ABLLS - Spontaneous Vocalizations): t(20)=-4.069, p<0.001, d=-0.888; Pair 19 (ABLLS - Syntax Grammar): t(20)=-3.008, p=0.007, d=-0.656; Pair 20 (ABLLS - Social Interactions): t(20)=-5.519, p<0.001, d=-1.204; and Pair 21 (ABLLS - Generalized Responding): t(20)=-5.888, p<0.001, d=-1.285. 

More detailed paired t-test results for both VBMAPP and ABLLS are presented in Table 1 below.

**Table 1:** Paired t-test with VBMAPP and ABLLS. Results are represented as paired mean difference, standard deviation, standard error of the mean, confidence interval of the difference (lower and upper), t, degrees of freedom, p-value (two-tailed), effect size-Cohen's d, and confidence interval for effect size-Cohen's d. VBMAPP: Verbal Behavior Milestones Assessment and Placement Program; ABLLS: Assessment of Basic Language and Learning Skills; VPMTS: Visual Perceptual Skills and Matching-to-Sample; LRFFC: Listener Responding by Feature, Function, and Class

Paired t-test	Outcome measure	Paired mean difference	Standard deviation	Standard error of the mean	Confidence interval of the difference	Confidence interval of the difference	t	Degrees of freedom	P-value (two-tailed)	Effect size-Cohen's d	Confidence interval for effect size	Confidence interval for effect size
					Lower	Upper					Lower	Upper
Pair 1	VBMAPP - Mand PRE - VBMAPP - Mand POST	-1.37500	0.82916	0.23936	-1.90182	-0.84818	-5.745	11	<0.001	-1.658	-2.531	-0.756
Pair 2	VBMAPP - Tact PRE - VBMAPP - Tact POST	-1.16667	1.05169	0.3036	-1.83488	-0.49845	-3.843	11	0.003	-1.109	-1.822	-0.367
Pair 3	VBMAPP - Listener Responding PRE - VBMAPP - Listener Responding POST	-1.79167	1.40548	0.40573	-2.68467	-0.89867	-4.416	11	0.001	-1.275	-2.032	-0.487
Pair 4	VBMAPP - VPMTS PRE - VBMAPP - VPMTS POST	-1.41667	1.12479	0.3247	-2.13132	-0.70201	-4.363	11	0.001	-1.259	-2.012	-0.476
Pair 5	VBMAPP - Independent Play PRE - VBMAPP - Independent Play POST	-1.33333	1.37069	0.39568	-2.20423	-0.46244	-3.370	11	0.006	-0.973	-1.652	-0.265
Pair 6	VBMAPP - Social Play PRE - VBMAPP - Social Play POST	-1.62500	1.18944	0.34336	-2.38074	-0.86926	-4.733	11	<0.001	-1.366	-2.15	-0.552
Pair 7	VBMAPP - Motor Imitation PRE - VBMAPP - Motor Imitation POST	-1.70833	1.26954	0.36649	-2.51496	-0.9017	-4.661	11	<0.001	-1.346	-2.123	-0.538
Pair 8	VBMAPP - Echoic PRE - VBMAPP - Echoic POST	-0.20833	0.33428	0.09650	-0.42072	0.00406	-2.159	11	0.054	-0.623	-1.233	0.010
Pair 9	VBMAPP - Spontaneous Vocalization PRE - VBMAPP - Spontaneous Vocalization POST	-0.70833	0.78214	0.22578	-1.20528	-0.21139	-3.137	11	0.009	-0.906	-1.57	-0.213
Pair 10	VBMAPP - LRFFC PRE - VBMAPP - LRFFC POST	-0.45833	0.94046	0.27149	-1.05587	0.13921	1.688	11	0.119	-0.487	-1.078	0.123
Pair 11	VBMAPP - Intraverbal PRE - VBMAPP - Intraverbal POST	-0.87500	0.93237	0.26915	-1.4674	-0.2826	-3.251	11	0.008	-0.938	-1.610	-0.238
Pair 12	VBMAPP - Group Behavior PRE - VBMAPP - Group Behavior POST	-1.12500	1.31642	0.38002	-1.96141	-0.28859	-2.96	11	0.013	-0.855	-1.508	-0.174
Pair 13	VBMAPP - Linguistic Structure PRE - VBMAPP - Linguistic Structure POST	-0.62500	0.90767	0.26202	-1.20171	-0.04829	-2.385	11	0.036	-0.689	-1.309	-0.043
Pair 14	ABLLS - Receptive Language PRE - ABLLS - Receptive Language POST	-45.571	33.227	7.251	-60.696	-30.447	-6.285	20	<0.001	-1.372	-1.964	-0.762
Pair 15	ABLLS - Requests PRE - ABLLS - Requests POST	-25.0000	21.38	4.665	-34.732	-15.268	-5.359	20	<0.001	-1.169	-1.72	-0.602
Pair 16	ABLLS - Labeling PRE - ABLLS - Labeling POST	-37.14300	31.457	6.864	-51.462	-22.824	-5.411	20	<0.001	-1.181	-1.733	-0.611
Pair 17	ABLLS - Intraverbals PRE - ABLLS - Intraverbals POST	-34.0000	36.708	8.01	-50.709	-17.291	-4.244	20	<0.001	-0.926	-1.432	-0.404
Pair 18	ABLLS - Spontaneous Vocalizations PRE - ABLLS - Spontaneous Vocalizations POST	-7.28600	8.205	1.79	-11.02	-3.551	-4.069	20	<0.001	-0.888	-1.388	-0.373
Pair 19	ABLLS - Syntax Grammar PRE - ABLLS - Syntax Grammar POST	-11.762	17.922	3.911	-19.92	-3.604	-3.008	20	0.007	-0.656	-1.123	-0.177
Pair 20	ABLLS - Social Interactions PRE - ABLLS - Social Interactions POST	-22.28600	18.504	4.038	-30.709	-13.863	-5.519	20	<0.001	-1.204	-1.762	-0.63
Pair 21	ABLLS - Generalized Responding PRE - ABLLS - Generalized Responding POST	-7.76200	6.041	1.318	-10.512	-5.012	-5.888	20	<0.001	-1.285	-1.859	-0.694

## Discussion

The results of this study indicated statistically significant differences with all but two (11 out of 13) VBMAPP scales with moderate to mostly high effect sizes. The two non-significant VBMAPP scales were VBMAPP - Echoic (p=0.054, d=0.623) and VBMAPP - Listener Responding by Feature, Function, and Class (p=0.119, d=0.487). Note that these two scales have moderate effect sizes as measured by Cohen's d. Given that the sample size was relatively small (n=11) for VBMAPP, these non-significant p-values may be attributed to low statistical power due to a relatively small sample size.

Considering that a total sample size of 23 individuals with autism would be needed to attain a high effect size (0.80) for the difference between two dependent means [1] with a nominal alpha (α)=0.05 using a two-tailed paired t-test, this idea is plausible. Notable differences were observed across all ABLLS scales, with effect sizes ranging from medium to mostly high.

It is essential to subject ABA in DTT, mass trials, and NET to longer-term outcome studies documenting the benefits of discrete trial teaching in its more traditional context [10,11]. Peterson et al. [12-14] reported results for the ongoing impact of ABA using DTT and mass trials within a naturalistic environment with autistic individuals using repeated measures analyses. Statistically significant mean differences in target behaviors were delineated. Studies measuring and assessing the direct impacts of ABA on verbal behaviors, specifically using VBMAPP and ABLLS as outcome instruments, continue to be scarce in the literature, but new studies are germinating. 

VBMAPP

With the VBMAPP, manding skills significantly increased after the ABA treatments, suggesting an increased ability to request by asking and becoming more empowered to advocate for their wants and needs. Tacting skills were significantly increased, suggesting that the individual's abilities to name entities, such as a tree or a dog, improved. This increases understanding of their world based on what they see, hear, and touch [3]. Statistically significant increases in the listener responding realm point toward an increased ability to respond to verbal stimuli from others and comprehend and react to spoken language. This is an essential skill for effective communication and social interactions.

Visual perceptual and matching-to-sample skills significantly increased, as did the ability to perform visual discrimination tasks. These skills are fundamental for understanding and interacting with the environment and recognizing similarities and differences between objects and pictures. The results indicated significant increases in an individual's ability to engage in independent play activities without direct interaction or guidance from others [3]. This helps individuals learn to manage their emotions, solve problems, entertain themselves, and contribute to social development. Significant increases were noted in the patient's abilities to engage in social play, as indicated by play and interaction with others. This skill is essential for developing social skills, understanding social cues, and building relationships. Significant increases in turn-taking, joint attention, sharing, and understanding social rules were noted. Motor imitation skills were significantly increased with the ability to imitate physical movements demonstrated by others. This is important for learning and social interaction and allows the individual to learn by observing and replicating movements made by peers, caregivers, or teachers [3].

Though statistically non-significant, children's ability to repeat, echo, or imitate spoken words after hearing them indicates a moderate effect size. Echoic behavior is a vital component of language development, which allows individuals to learn and practice verbal skills by imitating sounds and words. Statistically significant improvements were noted with spontaneous vocalization, an individual's ability to verbalize sounds or words without being directly prompted or imitating a peer or teacher. It involves spontaneously using language to express thoughts, feelings, or needs. Spontaneous vocalization is a critical language development accomplishment. It allows independent communication and sharing of their experiences with others [3]. Non-statistically significant differences were discovered with skills associated with listener responding by feature, function, and class, an advanced listener-responding behavior focusing on identifying objects based on specific characteristics. It involves recognizing objects by their associated features, functions, or categories. It teaches individuals adjectives and verbs related to different objects. However, a moderate effect size was observed.

ABA treatments significantly increased individual's interverbal skills. These abilities refer to responses to verbal stimuli from others without direct physical cues and involve developing novel responses within the proper context [3]. Group behavior skills significantly increased. The ability of autistic individuals to perform suitable behaviors in a group or classroom environment includes obeying group commands, cooperating in group tasks, and displaying social skills. These skills are essential for successful inclusion in educational and social environments, allowing individuals to interact with peers, teachers, and classmates effectively. Individual's linguistic structure abilities, as demonstrated by language complexity, grammatical skills, sentence structure, verb tenses, pronouns, and syntax, were significantly increased [3]. 

ABLLS

With the ABLLS, receptive language skills significantly increased, which points toward an individual's ability to understand and process spoken or written language. The individuals were better able to comprehend words, sentences, and instructions. Teachers and therapists model and prompt appropriate verbal behaviors, clearly presenting language examples and providing cues to guide correct responses [15]. Statistically significant increases were observed in individuals' abilities to request or ask for desired items or actions, using language to express needs, wants, and preferences. Teachers and therapists use modeling, demonstrating how to make requests, and prompting, providing cues or assistance to encourage accurate requests. Individuals' abilities to name or label objects, actions, and concepts significantly increased. This also involves an individual's expressive language skills to identify and describe items in the environment. Teachers and therapists use strategies such as demonstrating behaviors (modeling), identifying objects verbally, giving examples, enhancing responses to encourage more complete descriptions, and offering reinforcement for identifying entities correctly [15].

There is a statistically significant increase in an individual's abilities to utilize intraverbals, where an individual responds to verbal stimuli from others without direct physical cues. Unlike echoing or repeating, intraverbals involve generating novel responses based on context. Finishing sentences and answering questions and the ability to engage in conversations and discuss topics also increased. Individuals' abilities to spontaneously vocalize were also statistically significant, as their ability to produce verbal sounds or words without direct prompting or imitation increased. The individuals' abilities to spontaneously use language to express thoughts, feelings, or needs significantly increased. Teachers and therapists use strategies such as creating opportunities and providing situations where the children can verbally express themselves [15].

Individuals' abilities with syntax grammar were significantly increased with the ability to use correct sentence structure and grammar and understand and produce grammatically accurate sentences. Teachers and therapists use strategies such as modeling correct sentences and providing examples of proper sentence structure. Statistically significant increases were noted in abilities to interact and engage in socially appropriate behaviors and interactions. Skills related to communication, joint attention, turn-taking, and understanding social cues were enhanced [15]. Teachers and therapists use strategies such as modeling, demonstrating appropriate social behaviors and social stories, creating narratives to teach social rules and expectations, and rewarding positive social interactions. Generalized responding significantly increased as skills and behavior across different settings, people, and materials were improved. The patients were able to apply learned skills in various contexts beyond the initial teaching environment. This ensures that skills are not limited to specific situations. It promotes flexibility and adaptability in using acquired knowledge [14].

ABA and verbal behavior outcomes

Behavior analysis has already contributed substantially to the treatment of individuals with autism, and further gains can result from more use of Skinner's analysis of language in verbal behavior (1957) and the resulting conceptual and experimental work. It is well known that ABA focuses on developing each verbal operant (rather than on words and their meanings) and the independent training of speaker and listener repertoires [2,6]. Skinner's analysis of verbal behavior may also help parents and professionals make decisions regarding general instructional approaches for patients [1]. Carr and Firth [19] commented on ultimately moving toward the execution of larger-scale experimental evaluations to provide evidence to support Skinner's analysis of verbal behavior and the clinical methods derived from that account. Ongoing studies using standardized outcome measures such as VBMAPP and ABLLS will put researchers in a better position to ensure that treatment dissemination efforts and supporting empirical evidence are better correlated. 

Limitations

The results of this study are informative, but it has limitations. A convenience sample was used, and a threat to external validity exists with no ability to generalize beyond this particular sample. Threats to internal validity apply in pretest-posttest research designs of this type. History points toward extraneous variables not part of the study or any external events that may affect outcomes. Maturation involves age-related bodily changes and includes age-related physical changes that can occur with time, such as hunger, tiredness, fatigue, wound healing, surgery recovery, disease progression, etc. Testing relates to the notion that the test may affect the children's responses when tested again. These are less of an issue when the tests are routine. Instrumentation refers to any change in measurement ability, including any judge, rater, etc. Statistical regression is the tendency for individuals who score extremely high or low on a measure to score closer to the mean of that variable the next time they are measured on it. Selection refers to the potential bias in selecting participants who will serve in the experimental and control groups. Mortality refers to the differential loss of study participants, drop-out rate, or attrition [20]. These threats to internal and external validity are always present in repeated measures studies of this nature. In addition, the heterogeneity of ASD assumes comorbid factors such as attention deficit hyperactivity disorder (ADHD), depressive disorders, epilepsy, intellectual disability (ID), sleep disorders, sight/hearing impairment/loss, and gastrointestinal syndromes which may have influenced the study's outcomes. The prevalence of comorbid conditions can vary widely among individuals with ASD [21].

## Conclusions

This study discovered that the application of ABA treatments notably enhanced the outcomes of verbal behavior in a convenience sample of 33 individuals with ASD. Statistically significant improvements were observed in various VBMAPP scales, including Mand, Tact, Listener Responding, Visual Perceptual Skills and Matching-to-Sample, Independent Play, Social Play, Motor Imitation, Spontaneous Vocalization, Intraverbals, Group Behavior, and Linguistic Structure. However, the VBMAPP Echoic and Listener Responding by Feature, Function, and Class scales did not achieve statistical significance, even though their effect sizes were moderate. All ABLLS scales, namely, Receptive Language, Requests, Labeling, Intraverbals, Spontaneous Vocalizations, Syntax Grammar, Social Interactions, and Generalized Responding, showed statistically significant outcomes. Additional confirmatory studies are needed to validate the reliability of these initial findings.
